# Description of a new species, *Sillago nigrofasciata* sp. nov. (Perciformes, Sillaginidae) from the southern coast of China

**DOI:** 10.3897/zookeys.1011.57302

**Published:** 2021-01-18

**Authors:** Jia-Guang Xiao, Zheng-Sen Yu, Na Song, Tian-Xiang Gao

**Affiliations:** 1 Laboratory of Marine Biology and Ecology, Third Institute of Oceanography, Ministry of Natural Resources, Xiamen 361005, China Third Institute of Oceanography, Ministry of Natural Resources Xiamen China; 2 Fisheries College, Ocean University of China, Qingdao 266003, China Ocean University of China Qingdao China; 3 Fisheries College, Zhejiang Ocean University, Zhoushan 316022, China Zhejiang Ocean University Zhoushan China

**Keywords:** DNA barcoding, molecular phylogenetic analyses, morphology, swim bladder, taxonomy

## Abstract

A new *Sillago* species, the black-banded sillago, *Sillago nigrofasciata***sp. nov.**, is described based on 302 specimens sampled from the southern coast of China. Morphological comparisons have been conducted between the new species and ten other *Sillago* species. The results show that the new species is characterized by a black mid-lateral band below the lateral line when fresh; other characteristics are similar to those of *Sillago sihama* but subtle differences exist on the swim bladder between *Sillago nigrofasciata* sp. nov. and *S. sihama*. A detailed description and illustrations are provided for the new species. The validity of this new species is also supported by a genetic comparison using sequences of the mitochondrial cytochrome c oxidase subunit I (COI) gene.

## Introduction

The family Sillaginidae Richardson, 1846, commonly known as sand whiting or sand borer, is a small family of demersal marine fishes that primarily inhabit inshore waters with sandy substrates or estuarine areas of rivers throughout the Indo-West Pacific (IWP; McKay 1985; McKay 1992; Nelson et al. 2016). At present, it is generally agreed that this family consists of 36 species, among them, five new *Sillago* species were published successively after the overview of the FAO species catalogue (McKay 1992), including *S. caudicula* Kaga, Imamura & Nakaya, 2010; *S. sinica* Gao & Xue, 2011; *S. suezensis* Golani, Fricke & Tikochinski, 2014; *S. shaoi* Gao & Xiao, 2016; and *S. panhwari* Panhwar, 2018 (Kaga et al. 2010; Gao et al. 2011; Golani et al. 2014; Xiao et al. 2016; Panhwar et al. 2018).

Reliance only on morphology to identify fishes to the species level is challenging when the diagnostic characters are similar among related taxa. Species of the family Sillaginidae are easily identified due to similarity of shape and coloration pattern (Sano and Mochizuki 1984; McKay 1992). This external morphological similarity, however, has led to much confusion in their specific identification and many cryptic species have been concealed in the synonymy of those wide-ranging species (Cheng and Zheng 1987; Kwun and Kim 2010; Bae et al. 2013). As a widely distributed Sillaginidae species, *Sillago sihama* (Forsskål, 1775) exhibits many cryptic lineages across its Indo-West Pacific distribution (Cheng et al. 2020). In fact, these five recently identified *Sillago* species were all wrongly assigned to *S. sihama*.

The most important character commonly used to identify *Sillago* species is its swim bladder. McKay (1985) reported three subgenera of the genus *Sillago*: *Sillaginopodys* Fowler, 1933 (swim bladder reduced, no duct-like process); *Sillago* Cuvier, 1817 (swim bladder divided posteriorly into two tapering extensions, duct-like process present); and *Parasillago* McKay, 1985 (swim bladder with a single posterior extension and the duct-like process). McKay (1985) also described four species of the subgenus Sillago (*S. S. intermedius* Wongratana, 1977; *S. S. megacephalus* Lin, 1933; *S. S. parvisquamis* Gill, 1861; and *S. S. sihama* Forsskål, 1775). The presence of two posterior extensions of the swim bladder observed in five species suggested that they should belong to the subgenus Sillago. In addition, a redescription of *S. indica* McKay, Dutt & Sujatha, 1985, reassigns it to the subgenus Sillago (Kaga and Ho 2012). This subgeneric grading system is very useful in both classification and phylogenetic analysis. Sometimes, the swim bladders of some sibling species are very similar, making the identification of these species extremely difficult, and other evidence must be found. In the last decades, DNA barcoding has provided an independent means of testing the validity of existing taxonomic units, revealing cases of inappropriate synonymy and, consequently, the existence of numerous cryptic species (Hebert et al. 2003a, b; Burns et al. 2008; Locke et al. 2010; Gao et al. 2011). Cheng et al. (2020) performed a thorough phylogenetic analysis based on both morphological and genetic evidences. The results indicated that more cryptic species could be present in the family Sillaginidae, and there are at least eight clades within the *S. sihama* complex.

While undertaking a taxonomic review of the genus *Sillago* along the southern coast of China, we had an opportunity to examine 302 specimens collected from this northwest Pacific Ocean coastline. Based on morphological characteristics, those specimens were assigned to *S. sihama*; particularly, their swim bladders were very similar to those of *S. sihama* (McKay 1992: fig. 130, type locality Queensland). However, a high mean genetic distance was found between these sequences and those of *S. sihama* based on DNA barcoding sequences. In addition, morphological evidence indicated that they belong to an unrecognized species. Herein, we use molecular and morphological approaches to describe the new species, and reconstruct the relationships of the species in the genus. Our results confirm the genetic distinction of the know *Sillago* species and invoke the possibility of additional species of *Sillago*, which may be hiding in the *S. sihama* cryptic complex along the coast of China.

## Materials and methods

### Sampling

**Figure 1.**
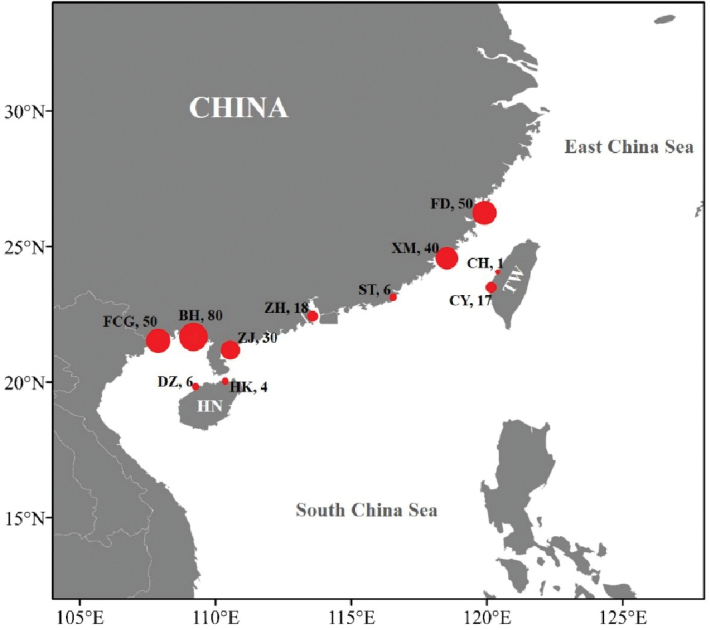
Sampling sites and corresponding sample sizes (represented by circle size and Arabic numerals) of *Sillago nigrofasciata* sp. nov. **HN**, Hainan Island; **TW**, Taiwan Island; **FD**, Fuding; **XM**, Xiamen; **ST**, Shantou; **ZH**, Zhuhai; **ZJ**, Zhanjiang; **BH**, Beihai; **FCG**, Fangchenggang; **DZ**, Danzhou; **HK**, Haikou; **CY**, Chiayi; **CH**, Changhua.

The unidentified specimens were collected from the southern coast of China, more precisely in Fuding (Fujian, 50 individuals), Xiamen (Fujian, 40 individuals), Changhua (Taiwan, 1 individuals), Chiayi (Taiwan, 17 tissues), Shantou (Guangdong, 6 individuals), Zhuhai (Guangdong, 18 individuals), Zhanjiang (Guangdong, 30 individuals), Beihai (Guangxi, 80 individuals), Fangchenggang (Guangxi, 50 individuals), Haikou (Hainan, 4 individuals), and Danzhou (Hainan, 6 individuals) (Fig. 1). All specimens were deposited at Fishery Ecology & Marine Biodiversity Laboratory, Fisheries College, Zhejiang Ocean University, Zhoushan (**ZJOU_FEBL**) and Fishery Ecology Laboratory, Fisheries College, Ocean University of China, Qingdao (**OUC_FEL**).

In this study, the recorded ten *Sillago* species with two posterior extensions of the swim bladder were referenced and compared to assign the new species (Table 1). Eight of them were used for genetic comparison altogether, including *S. indica*, *S. nigrofasciata* sp. nov., *S. panhwari*, *S. parvisquamis*, *S. sihama*, *S. shaoi*, *S. sinica*, and *S. suezensis*.

**Table 1.** Comparison of *Sillago nigrofasciata* sp. nov. and other ten species of *Sillago* with two posterior extensions of the swim bladder.

	*Sillago nigrofasciata* sp. nov.^a^	*S. intermedius* ^b^	*S. megacephalus* ^b^	*S. panhwari* ^a,c^	*S. shaoi* ^a,d^	*S. parvisquamis* ^a,b^	*S. sihama* ^a,d^	*S. caudicula* ^e^	*S. sinica* ^a,f^	*S. suezensis* ^g^	*S. indica* ^a,d,h^
**Dorsal fins**	X–XII, I, 20–22	XI, I, 21–22	XI, I, 22	X–XII, I, 20–22	XI, I, 20–22	XII–XIII, I, 20–22	XI, I, 20–23	XI, I, 22–23	X–XI, I, 20–22	X–XII, I, 19–22	X–XI, I, 20–22
**Anal fin**	II, 20–22	II, 21–22	II, 23	II, 18–23	II, 21–22	II, 22–24	II, 21–23	II, 23–24	II, 21–23	II, 18–22	II, 21–23
**Scales in lateral line**	67–75	67–70	70	69–84	70–73	79–84	68–72	71	75–79	63–74	68–71
**Scales above/below lateral line**	4–6/9–12	6–7/8–9	5/10–11	4–5/7–10	5–6/10–12	7/11–12	5–6/10–12	5/11	5–6/9–11	–	5–6/10–12
**Gill rakers first arch**	2–4/5–8	–	–	3–4/7–8	3–4/5–6	1–2/7–9	3/8–9	4/11	2–4/6–8	3–4/8–10	3–4/7–8
**Vertebrae**	34–35	34	–	34	35	39–40	34	35–36	37–39	34	33–35
**HL/SL (%)**	25.1–30.8	30.0–31.0	33.0	27.9–35.0	26.1–31.0	25.9–27.7	24.0–30.0	29.0–30.1	24.7–29.8	26.6–27.0	27.5–32.4

**Notes: a**, this study; **b**, McKay 1985, 1992; **c**, Panhwar et al. 2018; **d**, Xiao et al. 2016; **e**, Kaga et al. 2010; **f**, Gao et al. 2011; **g**, Golani et al. 2014; **h**, Kaga and Ho 2012.

### Morphological analysis

The genus and species classification followed McKay (1985), unless otherwise noted. The terminology of appendages of the swim bladder followed Shao et al. (1986) and Kaga and Ho (2012). In the descriptive section, the data of the holotypes were given first, while those of the paratypes followed in parentheses. General abbreviations used in this paper were:

**A** the number of anal fin rays;

**C** the number of caudal fin rays.

**D** the number of dorsal fin rays;

**HL** head length;

**P** the number of pectoral fin rays;

**SL** standard length;

**V** the number of ventral fin rays.

All measurements were made with dial calipers and dividers to the nearest 0.1 mm. The definition of the modified vertebrae followed McKay (1992). Gill rakers and swim bladders were examined in the dissected paratypes.

### Genetic analysis

To analyze genetic differences between this new species and other congeners, mitochondrial (mt) DNA cytochrome oxidase subunit I (COI) fragments of *Sillago* spp. were amplified based on the method of Ward et al. (2005). Genomic DNA extraction and polymerase chain reaction (PCR) followed the protocols of Gao et al. (2011). Sequences were checked and aligned using DNASTAR software (DNASTAR Inc., Madison, WI. USA) and MEGA 5.0 (Tamura et al. 2011) was used to analyze the sequences, estimate the pairwise genetic distances, and construct a Neighbor-joining (NJ) tree under the Kimura 2-parameter (K2P) model. COI sequences of *S. nigrofasciata* sp. nov. obtained in the present study were submitted to GenBank with the following accession numbers: KU051808, KU051809, KU051812, MG571453–MG571458, MG911029–MG911030. Twenty-nine COI sequences were obtained from GenBank with the following accession numbers: *S. indica* (KM250229–KM250232), *S. panhwari* (MF571945, MF571947, KU051787 and KU051788), *S. parvisquamis* (HQ389247–HQ389249), *S. shaoi* (KU051872, KU051873, KU051879, KU051886, and KU051887), *S. sihama* (KU051813, KU051819, KU051857, KU051803, and KU051881), *S. sinica* (KU052012, KU052017, KU052023, KU052025, and KU052029), and *S. suezensis* (FJ155362–FJ155364). *Sillaginodes punctata* was selected as the outgroup for genetic analyses based on a previous phylogenetic hypothesis of Xiao et al. (2016).

## Results

### Genetic analysis of the COI gene

**Figure 2.**
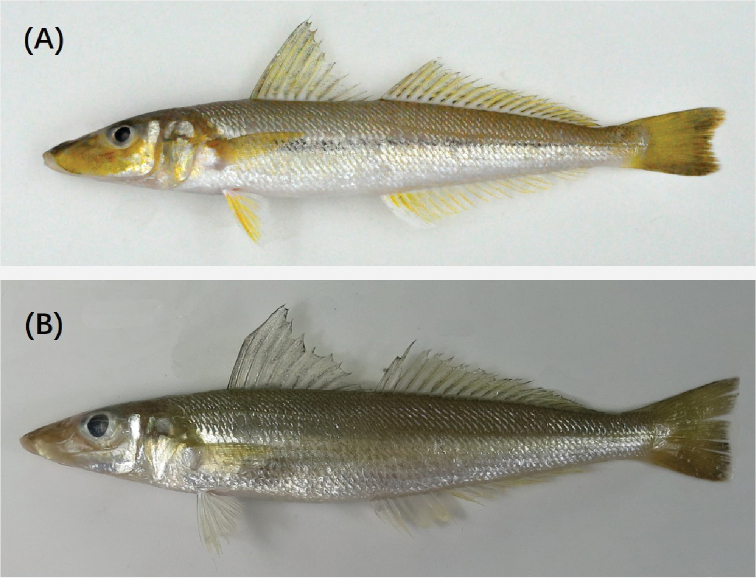
**A***Sillago nigrofasciata* sp. nov., OUC_FEL178001, holotype, 151.2 mm SL, Fuding, China, **B***Sillago sihama*, ZJOU_FEBL021131, 131.0 mm SL, Zhangzhou, China.

Forty specimens of eight *Sillago* species were used in the genetic analysis. There were no indels/insertions, and 185 variable sites were observed. Pairwise genetic distances (K2P) were shown in Table 2. Genetic distances among species ranged from 0.084 to 0.224, the intraspecific distances ranged from 0.000 to 0.004. The NJ tree based on the COI gene sequences revealed that all previously recognized and the newly discovered *S. nigrofasciata* sp. nov. individuals formed monophyletic groups (Fig. 2). Furthermore, a strong genetic divergence was detected between *S. nigrofasciata* sp. nov. and its plesiomorphic sister species *S. sihama*.

**Table 2.** Net genetic distances (K2P) within (on the diagonal) and between (below the diagonal) the eight *Sillago* species.

	*S. suezensis*	*S. parvisquamis*	*S. indica*	*S. sinica*	*S. sihama*	*S. shaoi*	*S. panhwari*	*S. nigrofasciata* sp. nov.
***S. suezensis***	0.000±0.000							
***S. parvisquamis***	0.193±0.021	0.000±0.000						
***S. indica***	0.084±0.012	0.202±0.022	0.002±0.001					
***S. sinica***	0.211±0.023	0.168±0.019	0.214±0.023	0.001±0.001				
***S. sihama***	0.177±0.020	0.210±0.022	0.172±0.020	0.211±0.023	0.001±0.001			
***S. shaoi***	0.213±0.023	0.152±0.017	0.224±0.023	0.124±0.015	0.196±0.021	0.003±0.002		
***S. panhwari***	0.192±0.020	0.214±0.022	0.206±0.022	0.217±0.022	0.210±0.022	0.222±0.023	0.004±0.002	
***S. nigrofasciata* sp. nov.**	0.204±0.022	0.194±0.021	0.216±0.022	0.192±0.021	0.181±0.019	0.196±0.021	0.198±0.021	0.001±0.001

### Taxonomic account

#### Family Sillaginidae Richardson, 1846

***Sillago* Cuvier, 1817**

##### Sillago nigrofasciata sp. nov.

Taxon classificationAnimalia

C4F30FDB-36D3-5C05-8432-8CE5FBE33F3C

http://zoobank.org/43E56C5F-C745-469E-9EE8-A221AEA5BFD5

**Figure 3.**
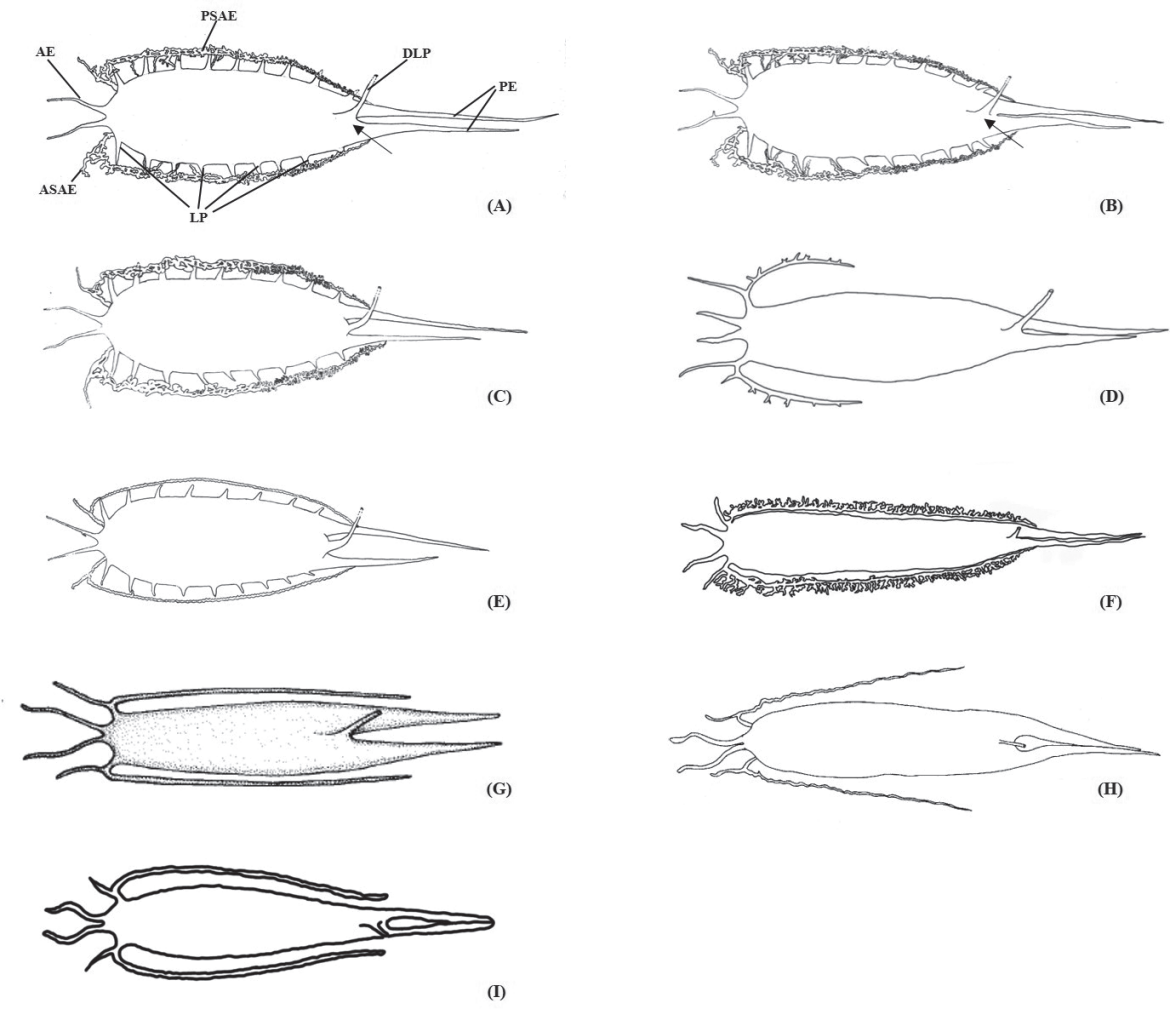
Swim bladders of nine *Sillago* species **A***S. nigrofasciata* sp. nov. **B***S. sihama***C***S. shaoi***D***S. sinica***E***S. indica***F***S. parvisquamis***G***S. intermedius* (McKay 1992) **H***S. caudicula* (Kaga et al. 2010) **I***S. suezensis* (sketch based on Golani et al. 2014). **AE**, anterior extension; **ASAE**, anterior sub-extension of anterolateral extension; **PSAE**, posterior sub-extension of anterolateral extension; **LP**, lateral processes; **DLP**, duct-like process; **PE**, posterior extension. Black arrows indicate differences between *S. nigrofasciata* sp. nov. and *S. sihama*.

**Figure 4.**
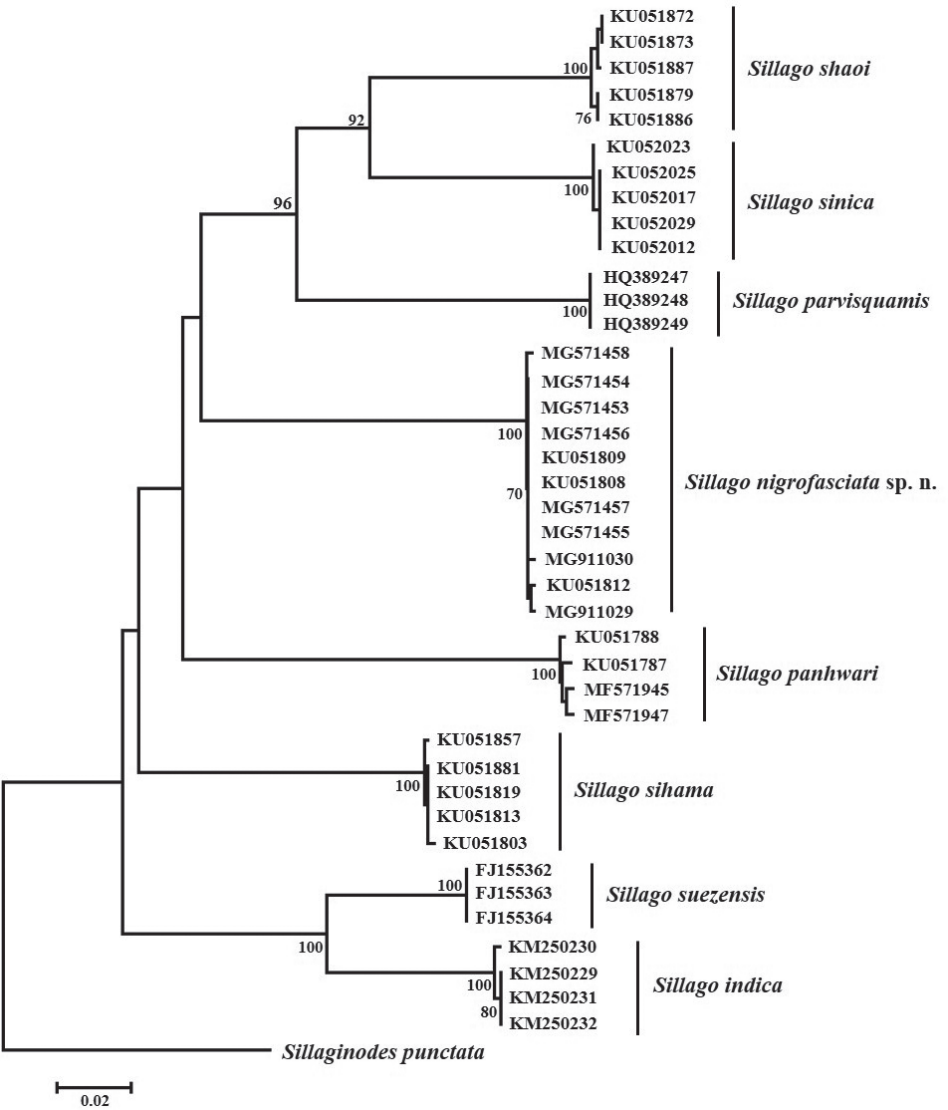
Neighbor-joining (NJ) tree for cytochrome oxidase subunit I (COI) gene sequences of eight species of *Sillago*. The NJ tree was constructed under the K2P model using *Sillaginodes punctata* as the outgroup. Bootstrap support values of > 70% from 1000 replicates are shown.

Figures 1 , 2 , 3 , 4 , Tables 1 , 2 , 3

###### Type material.

***Holotype*.** OUC_FEL178001, 151.2 mm SL, coastal area of Fuding, Fujian Province, China, collected by Yuan Li, January 2014.

***Paratypes*.** OUC_FEL178002–178030, 29 individuals, 134.4–161.4 mm SL, collection data same as for holotype; ZJOU_FEBL021255–021283, 29 individuals, 127.8–155.6 mm SL, coastal area of Xiamen, Fujian Province, collected by Jia-Guang Xiao, November 2015; ZJOU_FEBL021284, 1 individuals, 167.6 mm SL, coastal area of Changhua, Taiwan, collected by Shih-Chieh Shen, July 2014; OUC_FEL178051–178068, 18 individuals, 147.8–161.4 mm SL, coastal area of Zhuhai, Guangdong Province, collected by Bin-Bin Shan, December 2014; OUC_FEL178069–178098, 30 individuals, 120.3–163.0 mm SL, coastal area of Fangchenggang, Guangxi Province, collected by Dong-Ping Ji, December 2014.

###### Etymology.

The specific name *nigrofasciata* is a compound adjective derived from the Latin words referring to the wide mid-lateral black longitudinal band of this species, a diagnostic character of the species.

###### Diagnosis.

Relatively large body and usually with a wide mid-lateral black stripe from opercular to caudal peduncle; dorsal-fin rays X–XII (mostly XI), I+20–22, soft anal fin rays 20–22; scales in lateral line 67–75, scales above lateral line 4–6; gill rakers 2–4+5–8; vertebra: abdominal 14 or 15 (mostly 14), modified 3–7 (mostly 4 or 5), caudal 13–18, and total 34 or 35 (mostly 34) (Table 3). Swim bladder with two posterior extensions, the origin of the duct-like process at the terminus of swim bladder and start at the joint of roots of two posterior extensions (Fig. 4).

**Table 3.** Morphometric measurements for type specimens of *Sillago nigrofasciata* sp. nov.

Morphometric measurements (mm) and counts	Holotype	Paratypes (n = 107)
**Total weight (TW, g)**	37.0	16.2–49.3
**Total length (TL)**	174.5	140.4–187.8
**Standard length (SL)**	151.2	121.0–163.0
**Head length (HL)**	45.3	33.7–46.5
**Snout length (SL)**	18.9	15.0–22.3
**Eye diameter (ED)**	8.1	6.8–11.6
**Interorbital width (IW)**	10.7	6.0–14.1
**Postorbital length (PL)**	17.0	12.9–19.1
**Body depth (BD)**	24.3	17.4–27.2
**Body width (BW)**	19.3	17.0–22.4
**Length of caudal peduncle (LCP)**	16.2	10.8–20.0
**Depth of caudal peduncle (DCP)**	9.9	7.1–11.1
**Base of the 1^st^ dorsal fin**	36.7	25.3–40.0
**Base of the 2^nd^ dorsal fin**	52.6	41.1–64.8
**Base of the anal fin**	54.2	42.8–58.2
**Pectoral fin length**	24.2	18.2–27.1
**Ventral fin length**	23.1	17.4–27.2
**D**	XI, I+21	X–XII, I+20–22
**P**	16	14–16
**V**	I+5	I+5
**A**	II+22	II+20–22
**C**	17	16–18
**Gill rakers first arch**	3+7	2–4+5–8
**Vertebrae**	34	34–35
**Scales on lateral line**	69	67–75
**Scales above/below lateral line**	6/11	4–6/9–12
**As % of SL**		
**Body depth (BD)**	16.1	13.4–17.4
**Head length (HL)**	29.9	25.1–30.8
**Length of caudal peduncle (LCP)**	10.7	7.9–13.4
**As % of HL**		
**Eye diameter (ED)**	17.9	16.5–24.9
**Interorbital width (IW)**	23.5	14.8–31.2
**Snout length (SL)**	41.7	39.6–52.7
**Postorbital length (PL)**	37.6	33.2–42.4
**DCP/LCP**	61.0	51.3–88.5

###### Description.

General body features are shown in Figure 3. Counts and measurements are given in Table 3. Body elongate, anterior slightly pyramidal, posterior cylindrical; anterodorsal profile smooth. Body depth 16.1% (13.4–17.4%) in SL. Head large, length 29.9% (25.1–30.8%) in SL. Snout long, 41.7% (39.6–52.7%) of HL. Eye moderate, its margin slightly covered with adipose eyelid, diameter 17.9% (16.5–24.9%) of HL. Interorbital region flat, interorbital width 23.5% (14.8–31.2%) of HL. Nostrils situated anterior to upper margin of eye; posterior margin of anterior nostril with single anteriorly directed flap; posterior nostril lacking flap. Mouth small, terminal, anterior tip of upper jaw situated at almost same position as tip of lower jaw. Upper jaw with small canines forming a wide tooth band becoming narrower posteriorly. Lower jaw with small canines, forming tooth band anteriorly, width same as upper jaw tooth band, tooth band gradually becoming narrower posteriorly, ending in one row. Palatine and tongue toothless. Vomer with three to four rows of canine teeth. Posterior margin of preopercle slightly serrated. Gill aperture large, lateral, extending to ventral side of head, stopping at middle bottom of opercle. Gill rakers on the first arch pointed but short. Caudal peduncle short, depth of caudal peduncle 61.0% (51.3–88.5%) of length of caudal peduncle.

Body covered with small or moderate sized ctenoid scales, and cheek scales cycloid, arranged in two or three rows. Lower part of pre-opercular-mandibular canal covered with cycloid scales. The base of pectoral fin and ventral fin lacking scales. Lateral line beginning above gill aperture and anterior portion of pectoral fin, extending along curve of dorsal edge to the end of body.

Two separated dorsal fins, first dorsal fin XI (X–XII), obviously higher than second, origin posterior to top of pectoral fin base, composed of spines, gradually shortening. Fin membrane with dense black spots. Base of second dorsal fin long, composed of a single spine and 21 (20–22) soft rays, originating mid-body, and not extending to caudal fin origin when placed flat. Origins of anal fin slightly posterior to cloacal pore, with II+22 (20–22), not extending to caudal fin origin when placed flat. Pectoral fin 16 (14–16), slender. Two separated ventral fins broad, I+5, approximately triangular, and shorter than pectoral fin.

###### Color of fresh specimens.

Upper surface of head dark brown and trunk bright brown, grading to silver on abdomen. Dorsal side of snout brownish gray. Cheek yellow, slightly silver posteriorly, with black dots amassed on the anterior inferior part of eyes. A wide faint stripe composed of tiny black dots on skin always present, from opercular to caudal peduncle. Dorsal fins yellowish hyaline, small dark dense spots on fin membrane. Pectoral, ventral, and anal fins yellowish hyaline with dark spots; caudal fin yellowish dusky with a black margin and grayish brown margin posteriorly, lobes usually broadly truncated posteriorly.

###### Swim bladder.

Swim bladder large. Two anterior extensions diverging to terminate on either side of the basioccipital above the auditory capsule. Two posterior tapering extensions of the swim bladder penetrating into the caudal region, one usually longer than the other. Two anterolateral extensions originate anteriorly, each branch into anterior and posterior sub-extensions: the anterior one comprising a short, simple blind tubule and the posterior sub-extensions kinked, long and complex, extending along the abdominal wall ventral to the base of the posterior extensions, respectively, tangent but not interconnected. A single duct-like process originating from ventral surface of swim bladder extending to the urogenital opening and a sub-extension connecting with a sanguineous vesicle close to vertebra, of unknown function. Eight or nine lateral processes extending from entire lateral surface of main body of swim bladder, anterior three or four stout and horn-like, posterior five or six small and triangular in shape.

###### Habitat.

Habitat is similar to *S. sihama* in nearshore areas and frequently entering estuaries for considerable periods, it is common along the beaches, sand bars, and mangrove creeks with sandy substrates. Depths ranging from 0 to 20 m, and frequently captured by trawling vessels.

###### Distribution.

*Sillago nigrofasciata* sp. nov. was only found along the southern coast of China including the coastal waters of the South China Sea and the Taiwan Strait. Actually, its distribution range is similar to that of *S. sihama* in China (Fig. 1).

###### Comparisons.

According to the subgeneric grading system in *Sillago* proposed by McKay (1985), we used the characters of swim bladder, especially, the number of the posterior extensions, to divide *Sillago* into several categories. This study confirmed the validity of a new species with two posterior extensions by comparison-elimination with other species in the same category. Among the ten known members of *Sillago* with two posterior extensions, *S. nigrofasciata* sp. nov. was easily distinguished from *S. intermedius* and *S. caudicula* by the body coloration (dusky black blotches were present on the body of *S. intermedius* and *S. caudicula*), from *S. parvisquamis* and *S. sinica* by the dusky spots on the second dorsal fin membranes (five or six rows in *S. parvisquamis* and three or four rows in *S. sinica*). Empirically, *S. nigrofasciata* sp. nov. could also be distinguished from *S. sihama*, *S. indica*, *S. panhwari*, and *S. suezensis* by the coloration of anal fin (the anal fin of *S. nigrofasciata* sp. nov. was usually yellowish with sparse black spots, the anal fin of *S. indica* was yellowish brown, but the anal fin of *S. sihama*, *S. panhwari*, and *S. suezensis* were hyaline; on the other hand, there were more black dots on skin and fins of *S. indica* than on *S. nigrofasciata* sp. nov. when fresh).

Moreover, by the primary diagnostic features (Table 1), *S. nigrofasciata* sp. nov. was easily distinguishable from other species by the following: *S. megacephalus* by having a smaller head (25.1–30.8% SL in *S. nigrofasciata* sp. nov. vs. 33.0% in *S. megacephalus*) and less soft rays in anal fin (20–22 in *S. nigrofasciata* sp. nov. vs. 23 in *S. megacephalus*), from *S. parvisquamis* and *S. sinica* by having 34–35 total vertebrae (39–40 in *S. parvisquamis* and 37–39 in *S. sinica*), from *S. parvisquamis* and *S. sinica* can also by having 67–75 scales on lateral line (79–84 in *S. parvisquamis* and 75–79 in *S. sinica*), and from *S. caudicula* by gill rakers (4/11 in *S. caudicula*) and soft rays in anal fin (23–24 in *S. caudicula*).

As for the shape of swim bladder (Fig. 4), that of *S. suezensis* was always controversial (Kaga 2013). Based on its original description, the figures of the swim bladder (Golani et al. 2014: 418, fig. 4A–C) were stylized, lacking the details of those provided by McKay (1985, 1992) and Kaga and Ho (2012). However, the sequences of *S. suezensis* (Mediterranean population) and *S. sihama* (Hong Kong and southern Red Sea populations) showed a strong genetic divergence (Tikochinski et al. 2013). Here, those sequences were also cited to verify authenticity of *S. nigrofasciata* sp. nov. and dismissed *S. suezensis* (Fig. 2). The swim bladder of *S. panhwari* was described as having narrow anterior extensions joined at the origin, diverging to terminate on both sides of the basioccipital above the auditory capsule whereas the two posterior extensions penetrate into the caudal region, one usually longer than the other, and with a duct-like process (Panhwar et al. 2018). But based on the photo (Panhwar et al. 2018: fig. 3a), the swim bladder was flat and gasless, and the anterolateral extensions may be broken. Moreover, there was no description of the swim bladder of *S. megacephalus* by Lin (1933).

*Sillago sihama* was considered as having a wide Indo-Pacific distribution and consisting of more than one taxon. McKay (1992: 59, fig. 130) described two swim bladder patterns of *S. sihama* in the FAO Catalogue based on a Red Sea specimen and a Queensland specimen, with markedly different shapes and concomitant geographical divergence. The swim bladder of *S. nigrofasciata* sp. nov. was very similar to that of *S. sihama* and *S. shaoi*, but there were still some differences: the roots of two posterior extensions in *S. shaoi* were non-adjacent, the two posterior extensions were not well-knit in its natural state, and there was a lacuna between the two posterior extensions; the origin of the duct-like process was at the terminus of the swim bladder and between the roots of two posterior extensions. However, on the swim bladder of *S. nigrofasciata* sp. nov. and *S. sihama*, the roots of two posterior extensions were adjacent and two posterior extensions were in close proximity; and the difference between them was the origin of the duct-like process of *S. nigrofasciata* sp. nov. at the terminus of the swim bladder and starting at the joint of the roots of two posterior extensions, but the origin of the duct-like process of *S. sihama* was anterior to the terminus of the swim bladder and anterior to the joint of the roots of two posterior extensions. Moreover, the swim bladder of *S. indica* had the same framework as that of *S. nigrofasciata* sp. nov. excepting the thin simple anterolateral extensions (vs. *S. nigrofasciata* sp. nov., anterolateral extensions were twisted, long, and complicated). *Sillago nigrofasciata* sp. nov. could also be easily distinguished from *S. intermedius* and *S. caudicula* by those swim bladders with simple anterolateral extensions; *S. parvisquamis* stood out as having the strongest anterolateral extensions in comparison with the others (Fig. 4).

## Discussion

Species-level taxonomy for Sillaginidae species was mainly based on the external morphological characteristics and the shape of the swim bladder (McKay 1992; Kaga 2013). Differences between sibling species are generally small and restricted to only a few characters, most of which may be also subject to intraspecific variation. Furthermore, within several newly discovered species, morphological characters did not provide clear taxonomic resolution (Golani et al. 2014). This study described a new species, *Sillago nigrofasciata* sp. nov. As the name implies, the new species is characterized by the black mid-lateral band below the lateral line. However, in fact, there are a few *Sillago* species that have a black band along the sides, including *S. indica* and *S. parvisquamis*. These morphological similarities make identification difficult, especially in the complex *S. sihama* cryptic species group. The new research suggested that the *S. sihama* complex exhibited the highest level of genetic diversity, indicating that a series of *S. sihama* lineages were genetically represented at species level (Cheng et al. 2020). This study presented a thorough molecular phylogeny analysis of all species of *Sillago*, which were monophyletic with 100% bootstrap values. Specimens of *S. nigrofasciata* sp. nov. were grouped together, and shared significant genetic distances with *S. sihama* and other species in the COI genetic analysis. Genetic distances between *S. nigrofasciata* sp. nov. and other species ranged from 0.181 to 0.216, and the interspecific genetic distances were much greater than the intraspecific distances (0.000–0.004), which indicated that the COI gene used as a barcode is an effective tool to identify *Sillago* species.

According to the conventional classification of *Sillago* species, this species could be confused with *S. sihama* based on the countable characters and the shape of the swim bladder. In fact, as one clade of *S. sihama* complex (*S. sihama* ⑧ in Cheng et al. 2020), *S. nigrofasciata* sp. nov. were morphologically similar to *S. sihama* with little difference in swim bladder (Fig. 4), but with significant genetic difference (18.1%, Table 2). *S. nigrofasciata* sp. nov. clearly diﬀers from the true *S. sihama* by its distinct color pattern (with a wide faint stripe composed of tiny black dots on the skin from the opercular to caudal peduncle, and dark spots on the anal fin) and the swim bladder (the origin of the duct-like process of *S. nigrofasciata* sp. nov. is at the terminus of the swim bladder and starting at the joint of the roots of two posterior extensions). *Sillago sihama* lacks the mid-lateral stripe and dark spots on anal fin, and its origin of the duct-like process is anterior to the terminus of the swim bladder and anterior to the joint of the roots of both posterior extensions.

At present, the distribution of *Sillago nigrofasciata* sp. nov. overlaps with that of *S. sihama* in China: the coastal waters of the South China Sea and the Taiwan Strait. Actually, *S. sihama* across its Indo-West Pacific range exhibits by far the highest levels of genetic diversity. A few new species have been described in this range across the IWP: *S. caudicula* (from Oman and Madagascar), *S. sinica* (from China), *S. suezensis* (from the northern Red Sea and Mediterranean), *S. shaoi* (from Taiwan Strait), and *S. panhwari* (from the northern Arabian Sea), and they have been always regarded as junior synonyms of *S. sihama* (Kaga et al. 2010; Kaga and Heemstra 2013; Gao et al. 2011; Golani et al. 2014; Xiao et al. 2016; Panhwar et al. 2018). However, our DNA barcoding results indicate that there are more genetic lineages across the *S. sihama* range that probably represent species-level taxa. These findings indicate a thorough taxonomic review of *S. sihama* (and its junior synonyms) is necessary (Cheng et al. 2020).

## Supplementary Material

XML Treatment for Sillago nigrofasciata
